# A Combined Effect of G-Quadruplex and Neuro-Inducers as an Alternative Approach to Human Glioblastoma Therapy

**DOI:** 10.3389/fonc.2022.880740

**Published:** 2022-04-28

**Authors:** Galina Pavlova, Varvara Kolesnikova, Nadezhda Samoylenkova, Sergey Drozd, Alexander Revishchin, Dzhirgala Shamadykova, Dmitry Y. Usachev, Alexey Kopylov

**Affiliations:** ^1^ Laboratory of Neurogenetics and Genetics Development, Institute of Higher Nervous Activity and Neurophysiology of Russian Academy of Sciences (RAS), Moscow, Russia; ^2^ Federal State Autonomous Institution «N. N. Burdenko National Medical Research Center of Neurosurgery» of the Ministry of Health of the Russian Federation, Moscow, Russia; ^3^ Department of Medical Genetics, Sechenov First Moscow State Medical University, Moscow, Russia; ^4^ Chemistry Department, Lomonosov Moscow State University, Moscow, Russia

**Keywords:** glioblastoma multiforme, cancer stem cells, cell reprogramming, CD133, aptamers

## Abstract

Cancer cell reprogramming based on treatment with G-quadruplex, having antiproliferative power, along with small molecules able to develop iPSCs into neurons, could create a novel approach to diminish the chance of glioblastoma recurrence and circumvent tumor resistance to conventional therapy. In this research, we have tested several combinations of factors to affect both total cell cultures, derived from tumor tissue of patients after surgical resection and two subfractions of this cell culture after dividing them into CD133-enriched and CD133-depleted populations (assuming CD133 to be a marker of glioblastoma stem-like cells). CD133^+^ and CD133^−^ cells exhibit different responses to the same combinations of factors; CD133^+^ cells have stem-like properties and are more resistant. Therefore, the ability to affect CD133^+^ cells provides a possibility to circumvent resistance to conventional therapy and to build a promising strategy for translation to improve the treatment of patients with glioblastoma.

## Introduction

Glioblastoma (GBM) is one of the most severe forms of tumors. We have yet to discover an effective mode of treatment for this malignant brain tumor. All conventional modern treatments, such as surgery, radiotherapy, and chemotherapy, provide only a minor prolongation of the lifespan of a patient. Therefore, GBM therapy requires searching for more effective and novel approaches. Glioma is a very heterogeneous tumor with abnormal cell proliferation regulation; hence, differentiation should be the focus of this search (1). The heterogeneity of the GBM cell population makes the tumor *per se* unresponsive to various conventional therapies and supports tumor recurrence. Some cells of the tumor are sensitive to treatment, yet others appear resistant, and their sustained proliferation promotes further tumor growth (2).

It is critical to note that the main goal of all approaches for glioma treatment is either to cause tumor cell death or to induce a toxic effect, and yet these approaches have failed to solve the problem effectively. We consider an alternative paradigm of tumor treatment: in contrary to inhibit, the stimulation of “maturation”of tumor cells could deprive them of their proliferative potential. Despite the fiascos of so called ‘differential therapy’, such an idea is beginning to attract the attention of researchers, for example, in the cases of rhabdomyosarcoma, rhabdoid tumors (3), and leukemia (4). Dawson et al. (3) demonstrated that a combination of chemotherapeutic agent Vincristin and the myogenic differentiation inducers TPA and GSK126 resulted in the differentiation of cell lines RD and A-204. Han et al. (4) applied the AhR agonist, 6-formylindolo[3,2-b]carbazole, along with the FMS-like tyrosine kinase 3 inhibitor, gilteritinib, to stimulate the differentiation of leukemic stem cells (SCs).

According to a hypothesis of GBM origin, the tumor derives and develops from cancer stem cells (CSCs). Extracted and isolated CSCs are capable of forming a new tumor and modulating the microenvironment (5, 6). Understanding the properties of CSCs is a prerequisite to developing any effector for these cells, and for targeted cancer therapy development (7, 8). But despite intensive searching for various biomarkers of CSCs (9), correct identification of these cells remains obscure.

A primitive glance at the GBM tumor reveals two types of cells. Because of asymmetric division, CSCs produce the entire tumor cells. CSC begets CSC and cancer progenitor cells (CPCs) as daughter cells, which have an immature, undifferentiated phenotype. The tumor has about 5% of CSCs (10) because of its low proliferative activity. CSCs have elevated resistance to effectors like conventional therapeutic ones. In contrast, CPCs form the bulk of the tumor, they actively proliferate, and they are sensitive to both chemotherapy and radiation therapies.

CD133 is considered a marker of CSCs, though a controversial one. In this research, we do not strictly ascribe CD133 as a marker of CSCs we just accept that CD133^+^ cells are more resistant to the effectors. CD133 could be a marker of GBM CSCs (11, 12); it is also a surface biomarker of normal brain SCs (6).

CD133 is a pentaspan transmembrane glycoprotein of 865 amino acids (about 97 kDa, and larger if glycosylated), and it is on the cell surface. The expression of CD133 has been documented in adult SCs and CSCs/initiating cells from several diverse tissue and cancer types (4, 7). The glycosylated epitope CD133/1 is located at the extracellular loop/domain of the protein; this epitope is used to isolate pluripotent primitive SCs, containing neuronal and other SCs. Epitope mapping indicates that the AC133 antibody recognizes the CD133/1 epitope in the extracellular loop 2 (13). SCs with tumorigenic potential are found in CD133^+^ isolated cells from cancer specimen. CD133 antigen is important for identifying stemness in numerous human malignant tumors of both mesenchymal and epithelial origins (14–16).

The exact role of CD133 in SCs is unclear, although the protein has been studied for quite a long time. The CD133 function is associated with the maintenance of stemness (17). The surface CD133 of neural SCs is different from the CD133 of brain CSCs. Kemper et al. (13) demonstrated that the presence of epitope CD133/1 is a characteristic of CSCs only. Therefore, CD133^+^ SCs are the main risk factor for tumor development in the brain and other organs (18). A lowering of the CD133^+^ portion in the total cell culture decreases the ability of self-renewal and tumor potential (19). Therefore, the properties of CD133^+^ cells in GBM must be studied in more detail.

However, the overall data on the significance of CD133 are contradictory. For instance, some results question the correlation between CD133 expression and patient survival (20). Also, CD133 on theglioma cell surface was poorly immunoreactive for anti-AC133 antibodies (21). The amount of CD133 in the membranes of glioma cells varies during the cell cycle. An assumption that the detection signal for CD133/1 epitope linearly reflects the amount of CD133 protein could be a source of disagreement on CD133 vs CD133/1. Consequently, the readouts from CD133/1 assessments are often interpreted in terms of the CD133 protein (21).

The goal of this study is to answer two questions: (i) is it possible to reverse conventional ‘dead-therapy’ of glioma into ‘differential-therapy’ by applying a combination of proliferation-breakers and neuro-inducers? and (ii) if YES, how significant is the role of CD133^+^ cells in providing cell population resistance under the similar treatment, as well as their ability to preserve the “immature” state (22), while producing proliferation activity.

In this research, we have generated a combination of effectors for two-step treatment: firstly, to gently halt tumor cell proliferation, and secondly, to stimulate the maturation of tumor cells to finally stop growing. G-quadruplex crypto-aptamers were chosen as the first effector, and iPS neuro-inducers were chosen as the second one. As proliferation-breakers, two G-quadruplex pseudo-aptamers (GQ) have been chosen. Bi-HD1 and bi-(AID-1-T) have been shown to reduce glioma cell proliferation (23). As the neuro-inducers, a combination of factors that are applied to stimulate neural differentiation of iPSCs has been chosen. The factors are: SB431542 (SB hereinafter), purmorphamine (PRM), LDN-193189 (LDN), and Brain-Derived Neurotrophic Factor (BDNF). SB destabilizes signaling pathways that maintain cell pluripotency (24). PRM is commonly used as an analog of Sonic Hedgehog protein, which is essential for nervous system development (25). Therefore, SB and PRM, when applied to neural differentiation of iPSCs, affect different stages of neural “maturation” of progenitor cells. LDN is an inhibitor of Bone Morphogenetic Protein (BMP) signaling, used for neural induction together with SB. Oncogenesis influences BMP4 expression, influencing the proliferation of GSCs (26). BDNF is a neural inducer of the final stage of neural cell “maturation” (27). Altogether, the abovementioned factors drive the concordant process of neural differentiation, providing a novel approach for ‘differential therapy’.

## Materials and Methods

### Primary Cultures of Glioblastoma Cells

Cells were obtained from human glioblastoma tissues after tumor resection. Cell culture G01 was chosen for this research as it exhibits high expression of the CD133 cell surface marker. This culture was obtained by washing cells in Versen solution (Paneco, Russia), incubation with 0.25% trypsin-EDTA (Ethylenediaminetetraacetic acid) solution (Gibco, UK) at 37°C for 40 min, and disaggregation from tissue followed by centrifugation at 1,000 rpm for 5 min. Cells were cultivated in DMEM/F12 (Dulbecco’s Modified Eagle Medium/Nutrient Mixture F-12) medium (Gibco, UK) containing 1% L-glutamine (Paneco, Russia), 10% FBS (Fetal Bovine Serum) (Thermo Scientific, USA), and 1% HEPES (4-(2-hydroxyethyl)-1-piperazineethanesulfonic acid) (Sigma-Aldrich, USA) at 37°C with a humidified atmosphere of 5% CO2. Passaging was performed at 80% confluency, for which cells were washed in PBS (Phosphate Buffered Saline) (Gibco, UK) and incubated with a 0.25% trypsin-EDTA solution. After trypsin inactivation by fresh medium, cells were centrifuged at 1,000 rpm for 5 min, the supernatant was removed, fresh medium was added, and the cells were resuspended and plated into cultural flasks. The cell count was defined using Trypan Blue 1:1.

### Cell Separation

A separation was performed following the CD133 MicroBead Kit-Tumor Tissue protocol. Before the separation, cells were passed through 40 μm filters and centrifuged at 1,000 rpm for 5 min. During the first step, the cells were labeled with CD133 Microbeads (Miltenyi Biotec, Germany). Cells were centrifuged at 1,000 rpm for 10 min, after which the supernatant was removed. The cell pellet was resuspended in 60 μl of buffer (PBS with 2 мМ EDTA and 0.5% BSA (Bovine Serum Albumin) (Amresco, USA)). We then added 20 μl of FcR Blocking Reagent to the suspension. After this, 20 μl of CD133 MicroBeads were added and resuspended. The mixture was placed at 4°C for 30 min and then 2 ml of the same buffer was added to the cell suspension, resuspended, and centrifuged at 1,000 rpm for 10 min. The supernatant was removed and 500 μl of fresh buffer was added and resuspended.

For magnetic separation, MS Columns (Miltenyi Biotec, Germany) were used. After placing a column in the magnetic field of a MACS Separator (Miltenyi Biotec, Germany) and washing the column with 1 ml of buffer, the cell suspension was applied to the column. The unlabeled cells that passed through the column were obtained in the first collecting tube. The column was washed 3 more times in the second collecting tube to remove unlabeled cells. The fractions of unlabeled cells were combined in one collection tube. In another collection tube, the magnetically-labeled cells were eluted 3 times by pipetting 1 ml of buffer and firmly pushing the plunger into the column.

Noted that because the CD133 marker is believed to represent stem cells that divide asymmetrically, there will always be a few CD133^−^ cells in CD133^+^ cultures.

### Cell Cultivation With Aptamers and Factors of Neurodifferentiation

In the first stage of the experiment, we used small molecules SB41542 (10 μM) (Miltenyi Biotec, Germany) and PRM (2 μM) (Miltenyi Biotec, Germany). The third factor used for this investigation was the neurotrophic factor BDNF (Miltenyi Biotec, Germany) at a concentration of 20 ng/ml. In the second stage of the experiment, LDN-193189 (1 μM) (Miltenyi Biotec, Germany) was added to the medium.

Two aptamers, bi-HD1 and bi-(AID-1-T), were used in the experiments. They were added to the medium to a final concentration of 37.5 μM. Before adding them to the cell cultures, aptamers were pre-formed at 95°С and cooled overnight at 4°С. During the first step, G01-derived CD133^−^ and CD133^+^ cells were treated with DNA aptamers, and after two days, one of the three neuro-inducers was added to the medium. When combinations of aptamers and small molecules were tested on G01 CD133^−^ and G01 CD133^+^ cells, the neuronal inducers were consistently added to the growth medium in several combinations (**Table 1**) by the following scheme: Two days after the addition of SB431542, PRM, or BDNF, or a combination of SB431542+LDN-193189 were added to the medium; on day 5, after partially changing the growth medium in all flasks, Purmorphamine was added to the flasks with SB431542+LDN-193189; on day 7, BDNF was added to the flasks with SB431542+LDN-193189+Purmorphamine.

**Table 1:** The combinations of the factors added to the G01 CD133^+^ and G01 CD133^−^ cell cultures.

Flask №.	G01 CD133^+^ culture	Flask №.	G01 CD133^−^ culture
1	Control (without bi-(AID-1-T))	1	Control (without bi-(AID-1-T))
2	bi-(AID-1-T)	2	bi-(AID-1-T)
3	bi-(AID-1-T) + SB431542	3	bi-(AID-1-T)+SB431542
4	bi-(AID-1-T) + Purmorphamine	4	bi-(AID-1-T) + Purmorphamine
5	bi-(AID-1-T)+BDNF	5	bi-(AID-1-T)+BDNF
6	bi-(AID-1-T) + SB431542, LDN-193189, Purmorphamine, BDNF	6	bi-(AID-1-T) + SB431542, LDN-193189, Purmorphamine, BDNF

No additional factors were added to the control flasks. The proliferation activity of cells was tested using the MTT assay 10 days after all exposures. Proliferation activity and gene expression (using the RT-PCR method) were also analyzed 20 days after all exposures. After testing, the most effective combinations of G01 CD133^+^ and G01 CD 133^−^ cultures were tested on G01 culture according to the same scheme.

### Cultivation of Neuro-Spheres

Cells were seeded in 12-well plates in a serum-free culture medium containing DMEM/F12 medium, 1% glutamine, Antibiotic–Antimycotic (Gibco, UK) and supplements B27 and N2 (2 ml per 100 ml of medium) (Gibco, UK), and FGF and EGF (20 ng/ml) (PeproTech, USA).

### Immunocytochemistry

Cells at a concentration of 1 × 10^4^ cells/well were cultivated in duplicate in 4-well plates for 24 h in 500 μl of DMEM/F12 medium with 1% glutamine and 10% FBS. After washing twice in PBS (pH 7.3), cells were fixed in 500 μl of 4% paraformaldehyde solution for 30 min at 4°C. Cells were then washed twice more in PBS (pH 7.3).

Staining was performed using the following primary antibodies: rabbit polyclonal Anti-CD133 antibody (dilution 1:20, #Ab16518, Abcam, UK), goat polyclonal Anti-CXCR4 antibody (dilution 1:100, #Ab1670, Abcam, UK), rabbit polyclonal Oct4 antibody (dilution 1:100, #Ab19857, Abcam, UK), rabbit polyclonal Nestin antibody (dilution 1:200, #AB5922, Chemicon, USA), goat polyclonal Sox2 antibody (dilution 1:100, #sc-17320, Santa Cruz, USA), mouse monoclonal Notch1 antibody (dilution 1:200, #MA1-91405, Invitrogen, USA), and chicken polyclonal Map2 antibody (dilution 1:500, #Ab5392, Abcam, UK). The primary antibodies were dissolved in PBS with 0.3% Triton X100 (Sigma-Aldrich, USA) as a detergent and 2% donkey serum (Jackson Immunoresearch, UK) and incubated for 2 h at room temperature. A solution with 1% FBS and 2% donkey serum was used as a negative control.

After being washed three times for 5 min in PBS (pH 7.3), cells were incubated for 1 h with the following secondary antibodies: donkey anti-rabbit antibodies conjugated with DyLight-488 (dilution 1:100, #711-545-152, Jackson Immunoresearch, UK), donkey anti-goat antibodies conjugated with Alexa Fluor 594 (dilution 1:100, #705-585-147, Jackson Immunoresearch, UK), and goat anti-chicken IgY H&L (Alexa Fluor 488) (dilution 1:100, #Ab150173, Abcam, UK). Then, cells were washed in PBS (pH 7.3) and stained with bisbenzimide (Sigma-Aldrich, USA) for 5 min at room temperature. After that, the cells were washed in PBS (pH 7.3), covered with glycerin, and analyzed by fluorescence microscopy. An Olympus IX81 (Olympus Corp., Japan) microscope was used for visualization with a computer-controlled motorized stage (Märzhäuser, Wetzlar) and an Olympus DP72 digital camera (Olympus, Münster, Germany).

After staining the G01 CD133^+^ and G01 CD133^−^ cell cultures with anti-Nestin, anti-Sox2, anti-Oct4, anti-Notch1, and anti-Map2 antibodies, we evaluated the intensity of cell fluorescence in the microscope field of view. After evaluating the brightness of the pixels on the micrographs, graphs were plotted expressing the ratio of the fluorescence intensity during cell cultivation to control.

### RT-qPCR

Expression of the following markers was measured in G01 CD133^+^ and G01 CD133^−^ cultures by RT-qPCR: CD133, DR4 and DR5, GFAP, Nanog, Oct4, Sox2, Notch2, L1CAM, Nestin, EGFR, Olig2, PDGFRa, and MELK.

For RNA isolation, the TRIzolTM Reagent (Sigma-Aldrich, USA) was used. DNA strand synthesis was performed using the MMLV RT kit (Evrogen, Russia). The initial G01 culture was used as a control. The expression was estimated at 7, 21, 35, and 42 days after the beginning of cell cultivation. The assay was performed under the following conditions: preliminary warming for 5 min at 90°C, denaturation for 10 s at 95°C, primer annealing and elongation for 30 s at 60°C. The number of cycles was set at 40. The primers used in the assay are presented in **Table 2**. The house-keeping genes used in the research were GAPDH, HPRT, and GUSB.

**Table 2:** Panel of primers for RT-qPCR.

Genes	Primers’ sequence, 5′ - 3′	Genes	Primers’ sequence, 5′ - 3′
EGFR	GTGACCGTTTGGGAGTTGATGA GGCTGAGGGAGGCGTTCTC	CD133	TGGATGCAGAACTTGACAACGT ATACCTGCTACGACAGTCGTGGT
Nanog	AATACCTCAGCCTCCAGCAGATG TGCGTCACACCATTGCTATTCTTC	PDGFRα	GGCATTCTTTGCAATACTGCTTAA CATCTGCCGATAGCACAGTGA
Oct4	CGAAAGAGAAAGCGAACCAG AACCACACTCGGACCACATC	DR4	AGGAGCCGGCAGATTTGACA GCATCAGAGTCTCAGTGGGGT
Sox2	ACACCAATCCCATCCACACT CCTCCCCAGGTTTTCTCTGT	DR5	GTT CCA GCC CTC CCT CAG AT GGT GCA AAT GAG ACT GCC CA
MELK	CAAACTTGCCTGCCATATCCT GCAAATCACTCCCTAGTGTGTT	L1CAM	CATGTGATGGAGCCACCTGT CCCAGCTCTTCCTTGGGTTT
Nestin	TTGCCTGCTACCCTTGAGAC GGGCTCTGATCTCTGCATCTAC	MAP2	CCAATGGATTCCCATACAGG TCCTTGCAGACACCTCCTCT
Notch2	GATCACCCGAATGGCTATGAAT CAATGCAGCGACCATCGTTC	HPRT	TGAGGATTTGGAAAGGGTGT GAGCACACAGAGGGCTACAA
GFAP	CTGCGGCTCGATCAACTCA TCCAGCGACTCAATCTTCCTC	GAPDH	AGATCCCTCCAAAATCAAGTGG GGCAGAGATGATGACCCTTTT
Olig2	CCAGAGCCCGATGACCTTTTT CACTGCCTCCTAGCTTGTCC	GUSB	CTTCTCTGACAACCGACGCC ACACCCAGCCGACAAAATGC

### Confocal Microscopy

Confocal microscopy was performed by transfection of cell culture HEK293 using TurboFect Transfection Reagent (Thermo Scientific, USA). After transfection, the cells were cultivated in 4-well chamber-slides for 48 h in a humidified atmosphere of 5% CO2. Cells were then washed three times in PBS with 1% BSA for 5 min. In the first three wells, 50 μl of CD133/2-PE antibodies (dilutions 1:12.5, 1:25, and 1:50, #130-113-748, Miltenyi Biotec, Germany) and anti-GFP antibodies (FITC) (dilution 1:200, # Ab6662, Abcam, UK) were added per well, while in the fourth well, 50 μl of CD133 MicroBeads (Miltenyi Biotec, Germany) with FcR Blocking Reagent and PBS c 1% BSA solution were added at a ratio of 3:3:44 accordingly. The chamber-slide was incubated for 15 min at 4°C in the dark, and then 300 μl of Hanks’ solution (Paneco, Russia) was added.

### MTT Assay

The proliferation of G01 CD133^+^ and G01 CD133^−^ cell cultures was determined by the MTT assay, and this was also used to detect changes in proliferation of cells treated with specific aptamers. Glioblastoma cells were seeded in 96-well plates at a cell density of 2,000 cells per well in 200 μl of culture medium DMEM/F12. Cells were cultivated for 48 h at 37°C in a humidified atmosphere of 5% CO2. They were then preformed at 95°C for 5 min in a water bath, and the oligonucleotides were then added. The cell medium was removed from the wells and aptamers were added in prepared concentrations. There were five replicates for each concentration. Cells were incubated for 72 h at 37°C in a humidified atmosphere of 5% CO2. After being removed, the medium cells were washed with PBS and cultivated for 24 h in 100 μl of culture medium. We then added 20 μl of MTS reagent (Promega, USA) to each well, and the plate was incubated for 2 h at 37°C in a humidified atmosphere of 5% CO2. Cells without aptamers were used as a positive control, and the cell medium was used as a blank. At λ = 490 nm, optical density was measured on a plate analyzer Tecan Infinite M200/Pro (Tecan, Switzerland).

### CD133 Insert Cloning

RNAzol ^®^ RT (Sigma-Aldrich, USA) was used to isolate total RNA from G01 cells. First, RNA strand synthesis was performed using an MMLV RT kit. To obtain the CD133 DNA fragment of interest—CD133fr—three consistent PCRs were run, followed by 1% agarose gel electrophoresis and DNA extraction using a QIAquick^®^ Gel Extraction Kit (Qiagen, Germany). A fragment of the CD133 protein from 485 to 865 amino acids was used as an insert. Primers were designed using NCBI (National Center for Biotechnological Information). Blast, melting temperature (Tm), and annealing temperature were estimated using the NEB Tm Calculator (New England Biolabs melting temperature calculator). The primers used in the assay are presented in **Table 3**. Annealing sites of the used primers on the target gene Prom1 are shown in **Supplementary Figure 1**.

**Table 3:** Panel of primers for RT-qPCR.

Primers	Primers’ sequence, 5′ - 3′
cd133 f2	CTGTTTATGTTAATAACACTGAA
cd133 f3	TGTTGGGTGCAGCAGGAAGAAA
cd133 r1	GCTTCTAGATCATATGCAAAT
cd133 r0	AATTCAAGGGGTCGATAATGTA
Prom1 r0	CATCAGCTATCAATGTTGTGAT
Xho1 f	TTTTCTCGAGCTTTCCTCATGGTTGGAGTT
BamH1 r	TTTTGGATCCTCAATGTTGTGATGGGCT

Q5TM High-Fidelity DNA Polymerase (Thermo Scientific, USA) was used for PCRs 1–3. We used 1% agarose gel electrophoresis in TAE (Trisacetate-EDTA) buffer with GeneRuler 100 bp Plus DNA Ladder (Thermo Scientific, USA) as a marker. The DNA concentration and purity were measured using NanoDrop 2000.

The first PCR (PCR1) was run using cd133 f2-cd133 r1 and cd133 f3-Prom1 r0 primer pairs. Amplification of the nucleotide sequence, corresponding to the second extracellular loop of CD133 (PCR2), was run using the cd133 f2-Prom1 r0 primer pair. For ligation with the peGFP-c1 vector, PCR3 was run using the XhoI f-BamH1 r primer pair. PCR amplification in all the reactions was carried out under the following conditions: Preliminary warming at 98°C for 30 s; denaturation for 10 s at 98°C; primer annealing for 15 s at 58°C for PCR1–2, and 51–58°C for PCR3; elongation at 72°C for 25 s in PCR1 and for 40 s in PCR2-3; and final elongation at 72°C for 2 min in PCR1 and for 5 min in PCR2-3. There were 30 cycles for PCR1 and PCR3, while for PCR2 there were 27.

The restriction of the insert (CD133fr) and peGFP-c1 vector was carried out by the XhoI and BamH1 sites of restriction. Ligation of CD133fr and peGFP-c1 vectors (**Supplementary Figure 2**) was performed using 50 μg of each with T4 DNA High Concentration ligase (New England BioLabs, UK), and the mixture was incubated overnight at 16°С.

The transformation of *Escherichia coli* TOP10 cells (Invitrogen, USA) with CD133fr/peGFP-c1 was carried out using heat shock. Screening of the grown colonies overnight after transformation with CD133fr/peGFP-c1 was performed using cd133 f3-cd133 r0 primer pairs with Taq-polymerase (5 units/μl) (Thermo Scientific, USA). PCR was carried out under the following conditions: preliminary warming for 30 s at 98°C, denaturation for 10 s at 98°C, primer annealing at 58°C for 15 s, elongation at 72°C for 15 s, and final elongation at 72°C for 2 min. There were 27 cycles. A probe with CD133fr/pGEM-T easy recombinant DNA was used as the positive control (intermediate results). Afterwards, a PCR was run using 1% agarose gel electrophoresis in TAE buffer with GeneRuler 100 bp Plus DNA Ladder. A probe corresponding to 552 bp was used for the experiments. This probe was shaken overnight in lysogeny broth (LB) medium at 37°С.

Bacterial DNA was isolated using the GeneJET Plasmid Miniprep (Thermo Scientific, USA). Sanger sequencing was conducted to verify the nucleotide sequence of DNA that corresponded to the second extracellular loop of CD133.

Glioblastoma cell transfection was performed using TurboFect Transfection Reagent (Thermo Scientific, USA). For confocal microscopy, human glioblastoma cells were seeded in a 4-well chamber slide and, following transfection, were incubated at 37°С in a humidified atmosphere of 5% CO2 for 48 h.

### Statistical Analyses

The imaging of fixed samples was performed on an inverted confocal microscope (Olympus IX81) with a computer-controlled motorized stage and an Olympus DP72 digital camera. A laser scanning microscope (Zeiss LSM 900) was used for confocal microscopy of live and fixed cells. Image analysis was performed using ZEN software and Microsoft Excel.

The MTT assay analysis was completed using i-control 1.10 software. A CFX96 Real-Time PCR System was used to measure the expression levels of target genes. Each sample was carried out in triplicate.

The concentration of samples obtained from DNA recombinant vectors was measured using a Nanodrop 2000 microvolume spectrophotometer for DNA samples.

Statistical analysis was conducted using GraphPad Prism 9. Data were presented as means ± SEM or means ± SD. Statistical significance was assessed using ANOVA, followed by Tukey’s *post hoc* test or Mann–Whitney U-test. In the figures, *p <0.05, **p <0.01, ***p <0.001, and ****p <0.0001.

## Results

### High Expression of CD133 in G01 Cell Culture From Patient Surgical Sample of GBM

**Figure 1:**
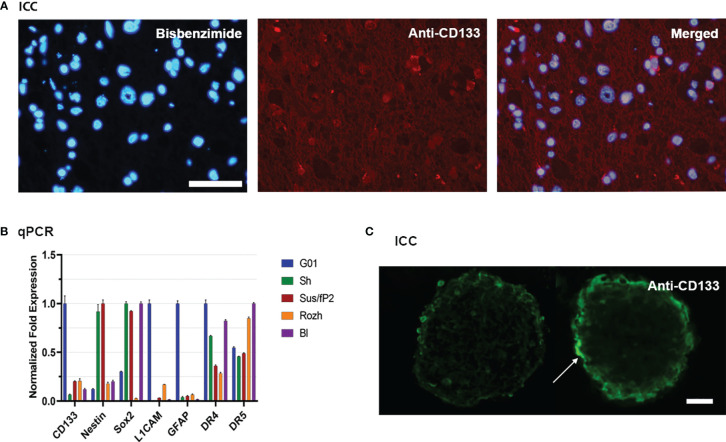
Characterization of glioblastoma cell culture with CD133 high expression rate. **(A)** Bisbenzimide staining of cell nuclei of the biopsy samples of the patient (left); immunocytochemical staining with anti-CD133 antibodies (middle); a and b merged (right); scale bar is 50 μm. **(B)** Real-time quantitative PCR. The expression of the genes of the neural stem cells in tested cell cultures. Data are represented as mean ± SEM; n = 3 for each group. **(C)** Immunocytochemical staining of the neuro-spheres with anti-CD133 antibodies. CD133^+^ cells (arrow) are located in the outer layer of the neuro-spheres; scale bar is 20 μm.

Because CD133 is the focus of this study, twenty cell cultures have been screened for this biomarker by real-time quantitative PCR (RT-qPCR). For further study, G01 cell culture has been selected because of the highest expression of CD133, which is about 10 times more than average (**Figure 1B**). A G01 cell culture has been developed from GBM tissue taken from a 37-year-old female patient with a tumor in the left frontal lobe. The initial tumor tissue transections have been tested by immunocytochemical staining with anti-CD133 antibody (**Figure 1A**), proving that initial patient sample is indeed enriched with CD133^+^ cells.

The reciprocal proves that it has also been performed. If G01 cell culture was cultivated in a serum-free medium, it actively developed neuro-spheres (**Figure 1C**), which have CD133^+^ cells.

Because further studies would be considered properties of SCs also, the transcription of some stemness genes has been measured by RT-qPCR for G01 cell culture, compared with other samples (**Figure 1B**). G01 cells have a low level of Nestin transcription, and a high level of transcription of L1CAM, CD133, and GFAP.

### Distribution of recCD133 in the Cell Membranes of G01 Cells Transfected With Recombinant DNA СD133CT/pEGFP-c1

**Figure 2:**
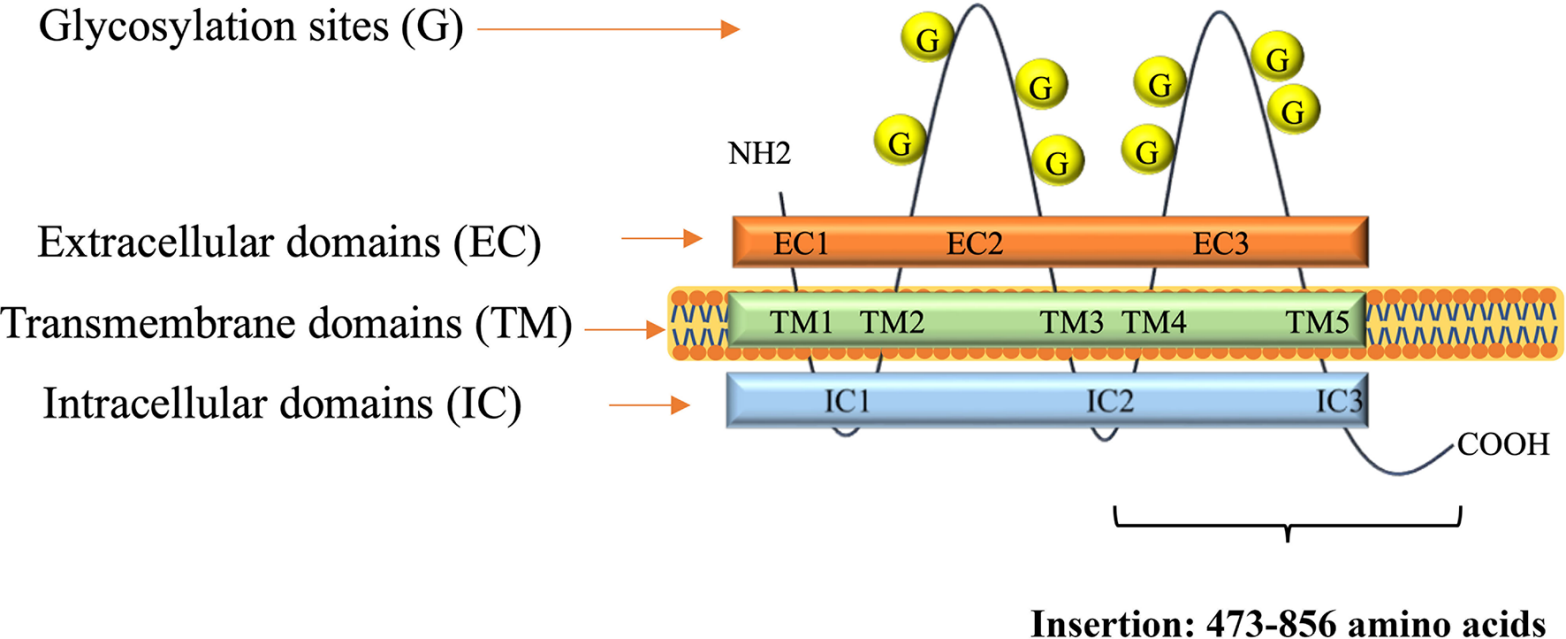
A scheme of CD133 protein structure. Insertion shows numbers of amino acids for CD133CT. The number of glycosylation sites is indicated schematically.

A DNA fragment of the CD133 gene: nucleotides 1694–2848, coding the C-terminal half of the protein CD133 amino acids 473–856, coined as CD133CT (**Figure 2**), has been cloned into a C-terminal protein fusion vector pEGFP-c1 after green fluorescent protein, GFP (**Supplementary Figure 2**). The construct GFP-CD133CT has been transfected into G01 cells. The following anti-CD133 antibody has been used: Miltenyi Biotech antibody clone AC133 recognizes epitope CD133/1, and antibody clone 293C3 recognizes epitope CD133/2. Both epitopes are in the extracellular loop/domain II of CD133. Abcam antibody Ab16518 binds to the C-terminal part of CD133 (28).

**Figure 3:**
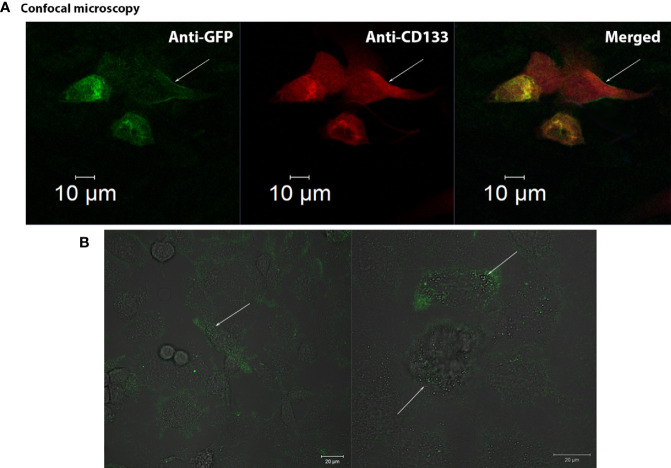
Confocal visualization of CD133 distribution on the cell membrane of G01 glioblastoma cells using СD133fr/peGFP-c1 recombinant DNA. **(A)** Micrographs of fixed glioblastoma cell culture cells stained with anti-CD133 antibodies (dilution 1:25) and anti-GFP antibodies (FITC) (dilution 1:200). GFP (green), CD133 (red), DAPI (blue). **(B)** Micrographs of the distribution of microbeads in glioblastoma cell culture transfected with реCD133fr/peGFP-c1. The arrows indicate magnetic beads. Scale bar is 20 mm.

CD133CT has both epitopes, CD133/1 and CD133/2, and it is built into the cell membrane (**Figure 3A**). A significant amount of protein was seen in the cell cytoplasm.

We also demonstrated that CD133 MicroBeads, having CD133/1 antibodies, bind to the transfected cells, proving that these microbeads could be effective in binding cells with the CD133 marker; the beads are located on the cell outer membrane (**Figure 3B**) (29).

We have demonstrated the membrane localization of CD133 with epitopes of interest using fusion proteins (**Supplementary Figure 3**). Anti-CD133/2-PE antibodies (#130-113-748, Miltenyi Biotec, Germany) effectively bind to CD133 chimeras on the outer membrane of the cell. A significant amount of protein was seen in the cell cytoplasm.

We have also examined the possibility of applying CD133-specific magnetic microbeads that will be used for isolating CD133^+^ cells and found that the beads are located on the cell outer membrane (**Figure 3B**) (29).

### Analysis of Cell Cultures G01 CD133^−^ and G01 CD133^+^

Using magnetic beads with immobilized anti-CD133/1 antibodies Miltenyi Biotec 130-100-857, from the initial G01 cell culture, we have isolated two cell fractions, coined as CD133^+^ and CD133^−^. It is worth keeping the following matter clear. Firstly, we understand that there is no solid evidence yet that CD133 is a genuine biomarker of either SCs or CSCs/GSCs. But if so, during G01 CD133^+^ culturing, a portion of CD133^+^ cells will be diluted because of an asymmetric cell division, and, actually, “CD133^+^” is “enrichment in CD133^+^.” Secondly, as we have also shown, for CD133^−^ it is unrealistic to completely deplete the cell culture of CD133^+^.

**Figure 4:**
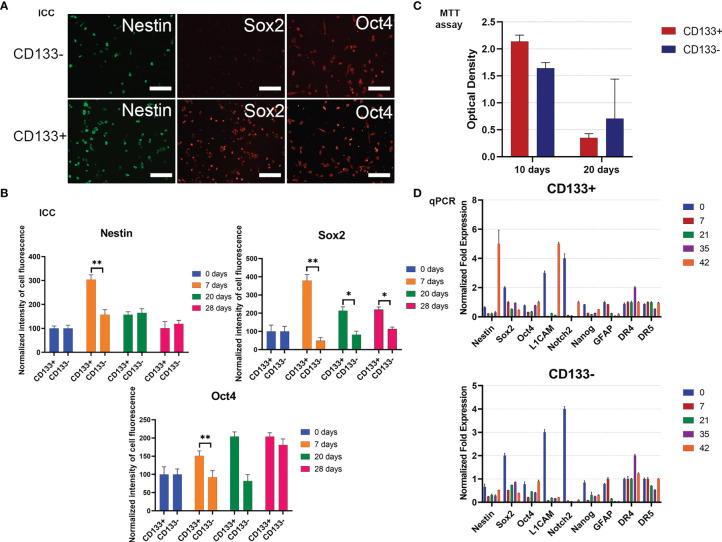
Characterization of G01 CD133^+^ and G01 CD133^−^ cell cultures after cell separation. **(A)** Micrographs of immunocytochemical staining of G01 CD133^+^ and G01 CD133^−^ cell cultures with anti-Nestin, anti-Sox2, anti-Oct4 antibodies. Scale bar is 200 μm. **(B)** The diagrams represent the normalized intensity of cell fluorescence of G01 CD133^+^ and CD133^−^ cell cultures. Data are represented as mean ± SD, n = 20 for each group. Statistically significant differences between the control and the treatment groups are indicated by asterisks (Mann–Whitney U-test, **p <0.01, *p <0.05). **(C)** MTT assay of G01 CD133^+^ and G01 CD133^−^ glioblastoma cell cultures in 10 and 20 days after cell separation; data are presented as mean ± SD; n = 5 for each group. Data are presented as mean ± SD. **(D)** Real-time quantitative PCR of stem cell genes in G01 CD133^−^ (left) and G01 CD133^+^ (right) cells before and at 7, 21, 35, and 42 days after cell separation. Data are presented as mean ± SEM; n = 3 for each group.

To begin with, the MTT test was used to estimate the proliferative activities of СD133^−^ and СD133^+^. Right after separation (zero time), then 10 days and 20 days after, the proliferation rate of СD133^−^ was higher than the rate of СD133^+^ (**Figure 4C**). After 30 days, both cell cultures had equal proliferation rates, and by day 70, CD133^−^ stopped proliferating and died, whereas CD133^+^ continued to grow with the same proliferation activity. Therefore, CD133^−^ has limited proliferation potential compared to CD133^+^.

To study how steady the expression of stemness genes is during prolonged culturing of both CD133^−^ and CD133^+^, the expression of some typical stemness protein markers has been measured by immunocytochemical analysis. By day 7, expression of Sox2 in CD133^+^ is about 7 times higher than in CD133^−^; this tendency slowly eases by day 28 (**Figures 4A, B**). By the same day, nestin expression in CD133^+^ is 2 times higher than in CD133^−^; the difference vanishes by day 20. There is no difference in the expression of Oct4. Expression of Map2 is the same in CD133^+^ and CD133^−^ (**Supplementary Figure 4**).

To extend the testing scale, the duration of culturing is increased to 42 days, with time intervals of 0, 7, 21, 35, and 42 days after the separation of the initial G01 cell culture. The stemness gene repertoire is also expanded.

Nestin, L1CAM, and Notch2 are the major biomarkers; other genes are GFAP, Sox2, Oct4, Nanog, and DR4, DR5. Nestin is a marker of neural stem cells (NSCs). L1CAM and CD133 are markers of glioma stem cells, GSCs (30). Fibrillary acidic protein (GFAP) is a marker for NSCs, GSCs, and mature glial cells (31).

For CD133^+^, transcription of genes exhibits intriguing features: by day 42, transcription of two major stemness genes, Nestin and L1CAM, had been lifted (**Figure 4D**). The same was true for Notch2, but to a lesser extent.

For CD133^−^, transcription of two major stemness genes, L1CAM and Notch2, was essentially decreased. The transcription of Sox2 was decreased 3–4 times by day 42 (**Figure 4D**).

### Treatment of G01 CD133^−^ and CD133^+^ With a Combination of Two Effectors: An Antiproliferative GQ and a Neuro-Inducer

Partial specification of G01 CD133^−^ and CD133^+^ has provided a prerequisite to initiate testing of a paradigm “first halt, then reprogram.” The following pairs of cell effectors have been chosen. To bein, two original GQs, bi-HD1 and bi-(AID-1-T) (23), were used to stop cell proliferation. Both GQs dose-dependently decrease cell proliferation rates (**Supplementary Figure 5A**). Additionally, GQs have some beneficial features: they reduce the proliferation of cancer cells cytostatically and they act transiently. Secondly, three well-known powerful neuro-inducers are used to reprogram cells: an inhibitor of activin receptor-like kinase receptors (SB431542, SB here), an activator of hedgehog signaling pathway (purmorphamine, PRM), and brain-derived neurotrophic factor (BDNF). All substances are widely used to stimulate neural differentiation of iPSCs (32).

**Figure 5:**
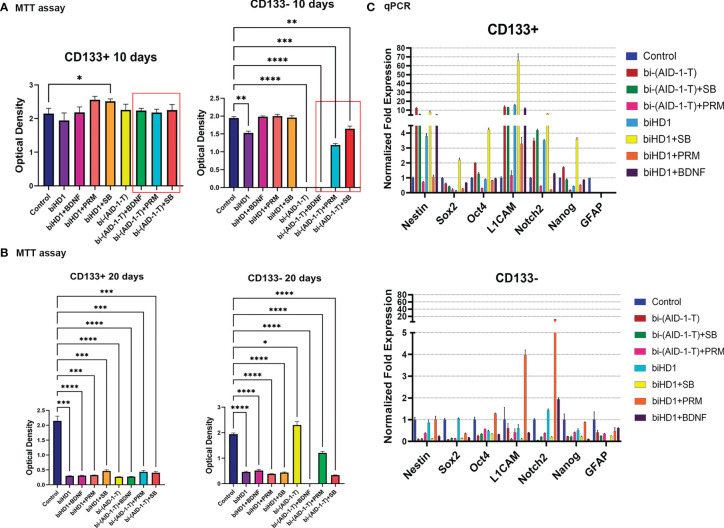
G01 CD133^+^ and CD133^−^ cells behavior after treatment of binary combination of GQ and a neuro-inducer. **(A)** MTT assay of G01 CD133^+^ cells (left) and G01 CD133^−^ cells (right) after 10 days treatment with combination of either biHD1 or bi-(AID-1-T) and neural differentiation inducers SB431542, purmorphamine, BDNF. Data are represented as mean ± SD, n = 5 for each group. Statistically significant differences between the control and the treatment groups are indicated by asterisks (One-Way ANOVA, *post-hoc* Tukey HSD Test, *p <0.05, **p <0.01, ***p <0.001, ****p <0.0001). **(B)** MTT assay of G01 CD133^+^ cells (left) and CD133^-^ cells (right) after 20 days treatment with the same binary combinations as in **(A)**. Data are represented as in **(A)**. **(C)** Real-time quantitative PCR of stem cell genes in G01 CD133^+^ (top) and G01 CD133^-^ (bottom) cell cultures in 20 days after the exposure to the aptamers biHD1 and bi-(AID-1-T) and neural differentiation inducers SB431542, purmorphamine, BDNF. Data are represented as mean ± SD. n = 3 for each group. SB, SB431542, PRM, purmorphamine.

For CD133^+^ any combination failed to change the proliferation rate for ten days. As expected, CD133^−^ are more sensitive to the treatment (**Figure 5A**). A very essential result is that bi-(AID-1-T) alone, and bi-(AID-1-T) with BDNF, [bi-(AID-1-T) + BDNF], significantly suppress cell proliferation. The cells stopped dividing and ceased culture growth. An apoptosis assay was performed to determine the percentage of apoptotic cells after culturing with the agents (**Supplementary Figure 6**). Two other GQ-low molecular effector combinations, [bi-(AID-1-T) + SB] and [bi-(AID-1-T) + PRM], had only minor effects.

After extending the treatment until day 20, the behavior of the cell cultures had changed dramatically (**Figure 5B**). For CD133^+^ cells, all treatments significantly reduced cell division. But for CD133^−^ some peculiar features have been found (**Figure 5B**). Cells were killed by the previously discovered effective combination [bi-(AID-1-T) + BDNF]. Interestingly, in the case of bi-(AID-1-T) alone, which was effective by day 10 and by day 20, the restoration of the ability of the cell to divide was observed, apparently because a GQ effect had ceased. In a similar vein, [bi-(AID-1-T) + PRM] had had a similar but minor effect. This finding supports the idea of using GQs as a transient effector to halt proliferation before the application of a neuro-inducer with [bi-(AID-1-T) + BDNF] being the most promising combination.

Both fractions, CD133^+^ and CD133^−^, treated for 20 days, had been analyzed for the transcription of stemness genes. When compared to CD133^−^, CD133^+^ showed significantly higher transcription of the major stemness genes L1CAM, Nestin, Notch2 (**Figure 5C**, top).

The results clearly indicate the possibility of combining two different effectors capable of finally reducing GSCs proliferation activity; the most promising combination is [bi-(AID-1-T) + BDNF].

### Treatment of G01 CD133^−^ and CD133^+^ With a Sophisticated Combination GQIcombi: Antiproliferative bi-(AID-1-T) Plus Cascade of Neuro-Inducers for iPSCs

**Figure 6:**
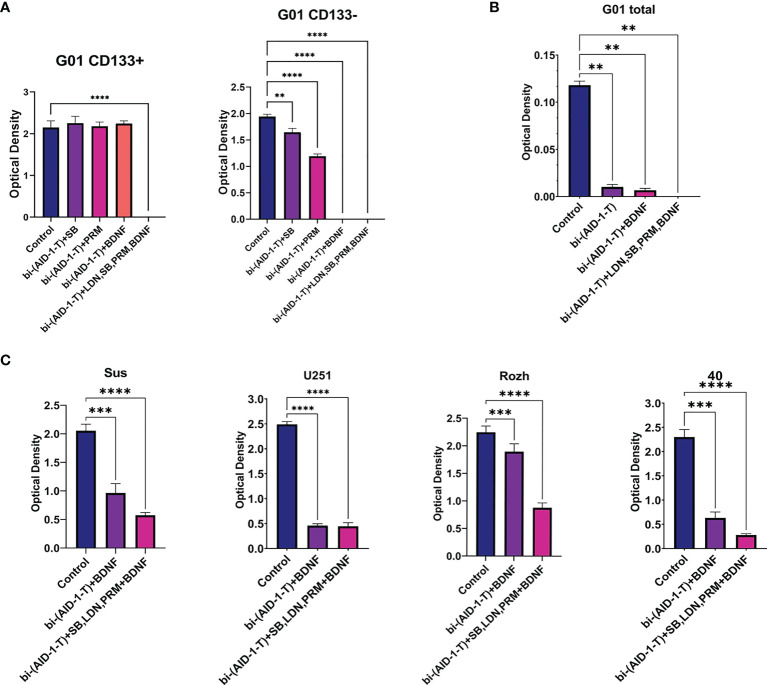
Exposure of cell cultures to the bi-(AID-1-T) aptamer and a cascade of neural inducers. **(A)** MTT assay for G01 CD133^+^ and G01 CD133^−^ in 10 days after the exposure to the bi-(AID-1-T) aptamer and the neural differentiation inducers. Data are represented as mean ± SD. n = 5 for each group. **(B)** MTT assay for G01 cells in 10 days after the exposure to the bi-(AID-1-T) aptamer and the successive addition of the neural differentiation inducers. Data are represented as mean ± SD. n = 5 for each group. **(C)** MTT assay for Sus, U251, Rozh and 40 cell cultures in 10 days after the exposure to the bi-(AID-1-T) aptamer and the neural differentiation inducers. Data are represented as mean ± SD. n = 5 for each group. Statistically significant differences between the control and the treatment groups are indicated by asterisks (One-Way ANOVA, *post-hoc* Tukey HSD Test, **p <0.01, ***p <0.001, ****p <0.0001). SB, SB431542; PRM, Purmorphamine; LDN, LDN-193189.

For CD133^−^, treatment with [bi-(AID-1-T) + BDNF] for 10 days was sufficient to block completely cell division, while for CD133^+^ cells any combination did not work (**Figure 5**). To enhance the effect of the neuro-inducer part of the combination treatment, instead of a single neuro-inducer, an original set of compounds, used for neural differentiation of iPSCs, has been applied. They are in use for SB, PRM, and BDNF, plus an additional new one, BMP signaling inhibitor LDN. They were added stepwise with a one-day interval. A panel of control experiments included initial cells and cells treated with either bi-(AID-1-T) alone or in combination with single effector from the GQIcombi. Proliferative activities of CD133^−^ and CD133^+^ were measured with the MTT assay after 10 days. Using GQIcombi, a complete stop of proliferation of CD133^−^, and more importantly, CD133^+^, has been observed (**Figure 6A**); a portion of **Figure 5A** in the red box is reproduced to demonstrate the effectiveness of [(AID-1-T) plus LDN, SB, PRM, BDNF]. In **Figure 6A**, we used the portion of **Figure 5A** highlighted in red to show the effect of [bi-(AID-1-T) + LDN, SB, PRM, BDNF] on the proliferative activity of CD133^+^ GBM cells. When all these influences are compared (either bi-(AID-1-T) plus one inductor or bi-(AID-1-T) plus all four inductors) (**Figure 6A**), the effect of bi-(AID-1-T) plus LDN, SB, PRM, and BDNF is clear. The neural marker bIII-tubulin was used to demonstrate an advanced differential state of G01 cells after exposure to GQIcombi (**Supplementary Figure 7**).

### GQIcombi Treatment of Several Glioma Total Primary Cell Cultures From Patients

Overcoming resistance of CD133^+^ with GQIcombi was a prerequisite to initiating testing of a paradigm “first halt, then reprogram” for the glioma total primary cell cultures from patients.

The initial G01 cell culture proliferation was inhibited more than 10 times with [bi-(AID-1-T) + BDNF] (**Figure 6B**); the inhibition was not complete, probably due to a presence of CD133^+^ fraction. However, the proliferation of the initial G01 cell culture was completely inhibited with GQIcombi (**Figure 6B**).

Taking into account the intertumoral heterogeneity of GBM, one more very essential matter is the variation in the response of different patient samples to the treatment. Indeed, the results of the treatment samples derived from different patients and the reference cell line U251 differ (**Figure 6C**), vary, but not in accordance with the previous observations. Some samples, such as Rozh, are rather resistant to treatment with just the binary combination [bi-(AID-1-T) + BDNF], but the GQIcombi is so effective that it successfully reduces the proliferation activity even in resistance cells.

## Discussion

To develop therapeutic approaches for treating a heterogeneous tumor population, one must first characterize tumor subpopulations and understand how these subpopulations could change during treatment. Molecularly defined, rare CD133^+^ cancer progenitor cells (CPCs) have been identified in a subset of gliomas and exhibit striking differences in response to treatment when compared to more differentiated tumor cells (33). Many tumors contain hierarchically organized cell subpopulations that retain the capacity to remake tumors and yet give differentiated tumor cell progeny. One might expect that selection would favor the evolution of tumors with high numbers of CPCs at the cost of differentiated cell types. Yet, paradoxically, in most malignancies, CPCs are far less abundant than differentiated cancer cells that cannot remake tumors. The changing of distribution of specific clones during tumor progression in the brain has also been observed (34).

To track the behavior of cell cultures and sub-populations, the following molecular biomarkers have been chosen. Nestin is one of the main biomarkers of neural stem cells, or NSCs (35). L1CAM is typical for NSCs and glioma CSCs (36). GFAP is a conventional biomarker of both NSCs and mature glial cells. GFAP is actively transcribed in NSCs, but in daughter progenitor cells its transcription is vanishes. Further transcription of GFAP re-appears in mature glial cells (31). Besides, GFAP is actively transcribed in glioma CSCs (31).

Notch2 was also included in the panel, as it could be a marker of NSCs (37). Notch2 is important in both carcinogenesis and the development of the most malignant gliomas (38). Enhanced Notch signaling correlates with a poor prognosis.

Other genes are the well-known Nanog, Oct4, and Sox2 (31, 39).

Current approaches to treating malignant tumors have different success rates. Gliomas are tumors that are still practically unamenable to therapy. The most aggressive and severe form of glioma is glioblastoma multiforme (GBM, Grade IV). Current therapy is limited to the surgical removal of the bulk of a tumor followed by radiotherapy (RT) and/or chemotherapy (CT), which is mainly based on toxic effects on cells. Drugs like Temodal kill proliferating tumor cells and healthy proliferating cells in the body.

Taking into account intratumor heterogeneity, simply consider two types of cancer cells: cancer stem cells (CSCs) and immature «daughter» cells of a tumor, which we coin cancer progenitor cells (CPCs) (40). Only CPCs actively divide and are targets for the RT/CT. On the contrary, CSCs, having relatively low proliferative potential, are rather insensitive to RT/CT; they cause a relapse later. Therefore, the very productive suggestion is not to kill actively dividing cells using death inducing drugs, but the other way around—to reprogram the cancer cells, CPCs, with differentiation inducers, change their differentiation status, and cease their proliferation.

The concept of ‘differentiation therapy’ was proposed in 1970s, and it was based on an idea that malignant cells could differentiate into less aggressive cells, therefore ‘differentiation therapy’ could be an alternative/complementary strategy to CT/RT-mediated cytotoxicity (41). However, with solid tumors this strategy has shown very limited success. Also, the strategy was not evaluated using a combination with conventional CT/RT, or with other effector of proliferation (42).

GBM can be considered a conglomerate of “daughter” tumor cells, CPCs, having a high, yet limited, ability to proliferate, and glioblastoma stem cells, GSCs, which maintain the whole population of GBM and supply its growth. Several studies have shown that GSCs are resistant to therapeutic agents (43, 44). Therefore, RT/CT will affect primarily CPCs, while GSCs survive and produce a new population (45).

Therefore, the idea of toxic affecting glioma cells should be reconsidered, and novel approaches for stimulation of tumor cell differentiation and ceasing their proliferation should be developed. Such approaches have been examined for some cancers. Xiong et al. considered tumor models as aggregates of CSCs and CPCs and explored the possibility of changing their differentiation potential (46). Han et al. reported a similar approach to leukemia treatment, where the aggression of cancer cells was reduced with a combined therapy for stimulating the differentiation of tumor cells (4). Dawson et al. (3) found that rhabdomyosarcoma and rhabdoid tumors harbor cells that are unaffected by chemotherapy and have properties similar to SCs. A three-drug combination of Vincristine with TPA/GSK126 differentiation therapy partially overcomes the chemoresistance of these cells.

This study searched for a combination of effectors able to stimulate neural differentiation of human GBM cells. There are GSCs in the heterogeneous population of glioma cells, and these cells are less mature than other cells in the tumor. CD133 biomarkers could be attributed to these cells. Mendiburu-Eliçabe et al. demonstrated that rapamycin significantly decreased the rate of cell growth, which correlated with a decrease in the expression of the CD133 gene of glioma CSCs in two cell cultures derived from GBM patients (47).

In this research, G01 cell culture derived from a human GBM patient has been studied. Initial G01 cell culture was split into two fractions: enriched with CD133 (CD133^+^) and depleted with CD133 (CD133^−^) (**Figure 4**). Following responses of these two separate fractions toward combinations of effectors that could stop proliferating, especially stem-like CD133^+^, enables the discovery of a very effective ‘GQIcombi’ combination.

The original idea for a treatment design was to select effectors able to decrease or halt cell proliferation for a while, and immediately after to stimulating tumor cell differentiation.

It is rather crucial that the first effector should not be quite toxic for cells, and it would only restrain the ability to divide of the cell. The unique GQ crypto-aptamer bi-(AID-1-T) has been chosen (23). CD133^−^ had stopped proliferating after 10 days of exposure to bi-(AID-1-T), and cell growth had recovered by day 20 (**Figure 5**). CD133^+^ had not been affected by this GQ (**Figure 5**).

As for the second effector, preliminary experiments with different substances that are strong neuro-inducers, like GDNF, IL6, and retinoic acid (RA) (**Supplementary Figures 5B, C**), did not provide the expected effect of proliferation arrest (48). Therefore, the molecules used for iPSC neural differentiation (49) have been applied. Firstly, we treated with SB, PRM, BDNF, one by one, after halting cell proliferation with bi-(AID-1-T). The application of BDNF alone, after GQ, was enough to block CD133^−^, but not CD133^+^ (**Figure 5**). This is in keeping with the data that CD133^+^ are immature cells, compared to CD133^−^, being SC-like; and BDNF is used for neural differentiation as a neurogenesis-encouraging factor applicable to CD133^−^.

Therefore, we used a stepwise treatment of CD133^+^ and CD133^−^ with the GQ and then with neuro-inducers according to the protocol of Wichterle and co-authors (50), modified by Lagarkova and co-authors (49).

For an effective treatment of CD133^+^, a short-term block of cell proliferation is required, with subsequent exposure to neuro-inducers, to manage the earliest stages of cell “maturation.” The molecule SB inhibits Lefty/Activin/TGFβ signaling pathways (41), which hinders differentiation and pluripotency. PRM is one more molecule affecting midbrain cell ‘ventralization’ (51). LDN is an inhibitor of theBMP signaling pathway (24). The last one, BDNF protein, is a neurotrophic factor that stimulates neuron maturation. It turned out that the exact combination of these substances was effective in blocking the proliferation of CD133^+^ cells (**Figure 6**). After a 10-day exposure, the cells stopped dividing and died. Therefore, for CD133^+^ cells, a step-by-step treatment is required, beginning from the earliest differentiation stages. It seems that the differentiation stage of CSCs is similar to embryonic SCs or induced SCs, and that could be a reason to apply a step-by-step treatment.

In summary, here we have demonstrated ‘proved-of-the-principle’ for a novel approach to inhibit glioma cell proliferation, which can change the state of tumor cells by stimulating their maturation, in contrast to conventional treatments that induce death. A combination of effectors has been discovered that could significantly inhibit the proliferation of primary GBM cells in patients. A very essential essence is that ‘GQIcombi’ blocks the division of CD133^+^ GSCs, which is crucial to overcome resistance, and to diminish or prevent tumor recurrence.

## Data Availability Statement

The original contributions presented in the study are included in the article/**Supplementary Material**. The raw gene expression data for this study can be found in the Harvard Dataverse (https://dataverse.harvard.edu/privateurl.xhtml?token=a795dbc7-0ef8-4a76-9988-51a97c5f2319). Further inquiries can be directed to the corresponding authors.

## Ethics Statement

The studies involving human participants were reviewed and approved by The Local Ethics Committee of Burdenko Neurosurgery Center. The patients/participants provided their written informed consent to participate in this study.

## Author Contributions

GP and VK contributed equally. Conceptualization, GP. Methodology, GP. Formal analysis, VK, NS, SD, AR, and DS. Investigation, VK, NS, SD, and AR. Resources, GP, DYU, and AK. Writing—original draft preparation, GP and VK. Writing—review and editing, GP and VK. Visualization, GP and VK. Supervision, GP and AK. Project administration, GP. Funding acquisition, GP. All authors listed have made a substantial, direct, and intellectual contribution to the work and approved it for publication.

## Funding

This research was funded by the Ministry of Science and Higher Education of the Russian Federation, grant number 075-15-2020-809 (13.1902.21.0030).

## Conflict of Interest

The authors declare that the research was conducted in the absence of any commercial or financial relationships that could be construed as a potential conflict of interest.

## Publisher’s Note

All claims expressed in this article are solely those of the authors and do not necessarily represent those of their affiliated organizations, or those of the publisher, the editors and the reviewers. Any product that may be evaluated in this article, or claim that may be made by its manufacturer, is not guaranteed or endorsed by the publisher.
