# Supplementary material for: A Combined Effect of G-Quadruplex and Neuro-Inducers as an Alternative Approach to Human Glioblastoma Therapy
Source: Front Oncol. 2022 Apr 28;12:880740. doi: 10.3389/fonc.2022.880740 (PMC9109612; doi:10.3389/fonc.2022.880740)
Supplement: Supplementary file 1 [file DataSheet_1.docx]

Supplementary Material


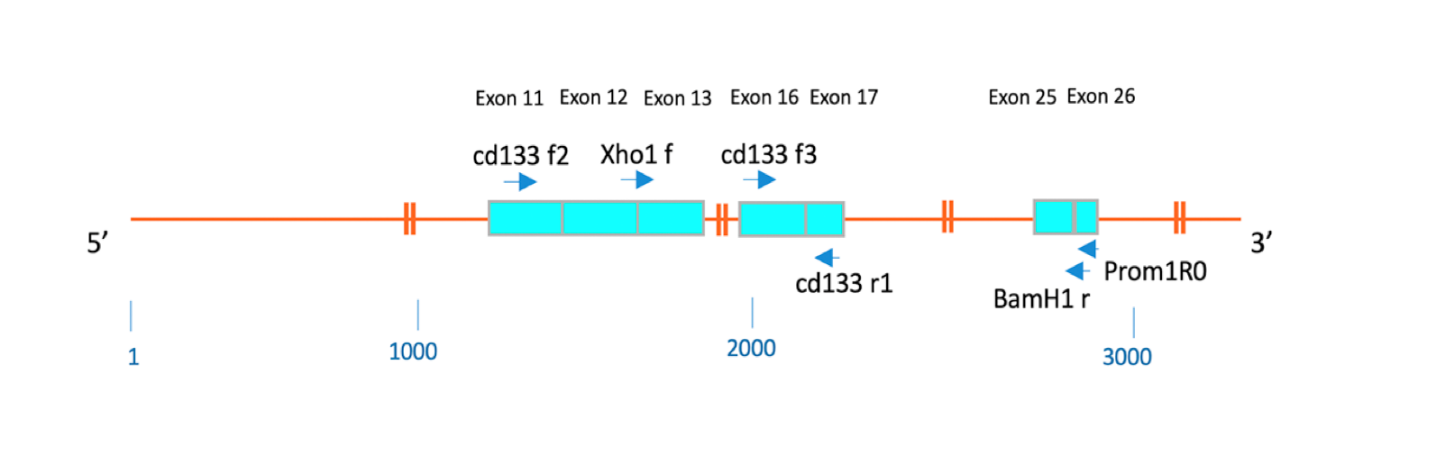


**Supplementary Figure 1.** Primer mapping on Prom1 gene. Human gene has 35 exons, and it is on the fourth chromosome 4p15.32 (Chr4: 15,968,226–16,084,059).


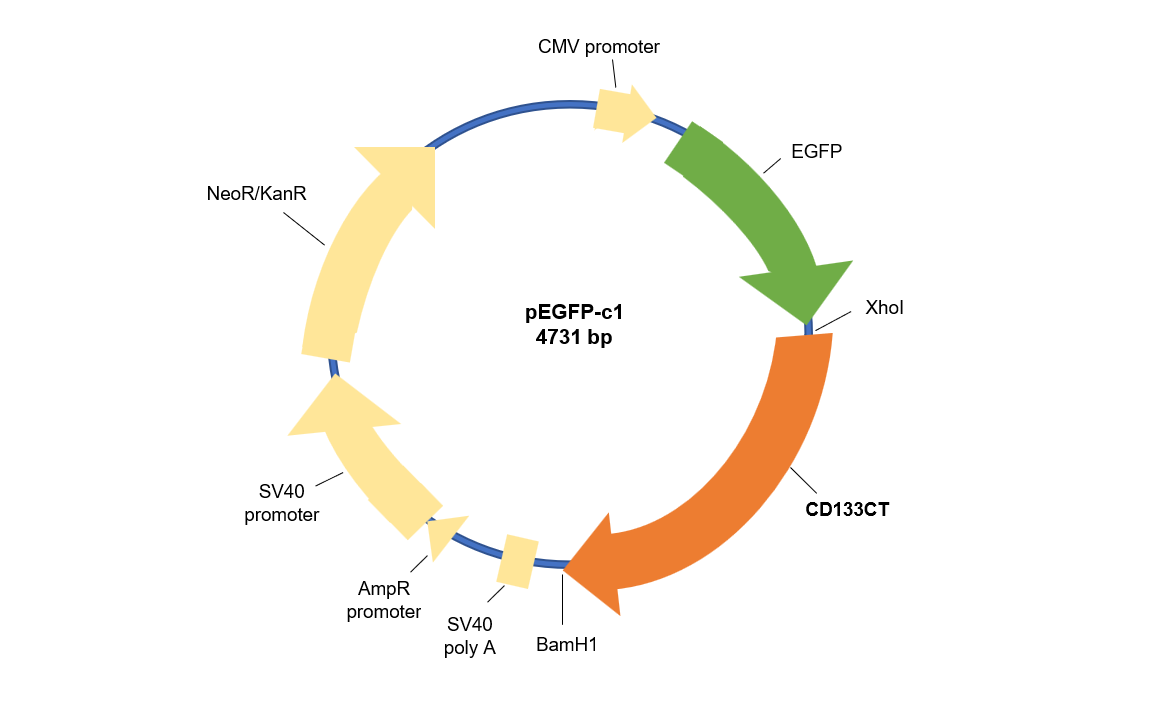


**Supplementary Figure 2.** The design of CD133CT/pEGFP-c1. The insert CD133CT is located between BamH1 and XhoI cites of restriction.


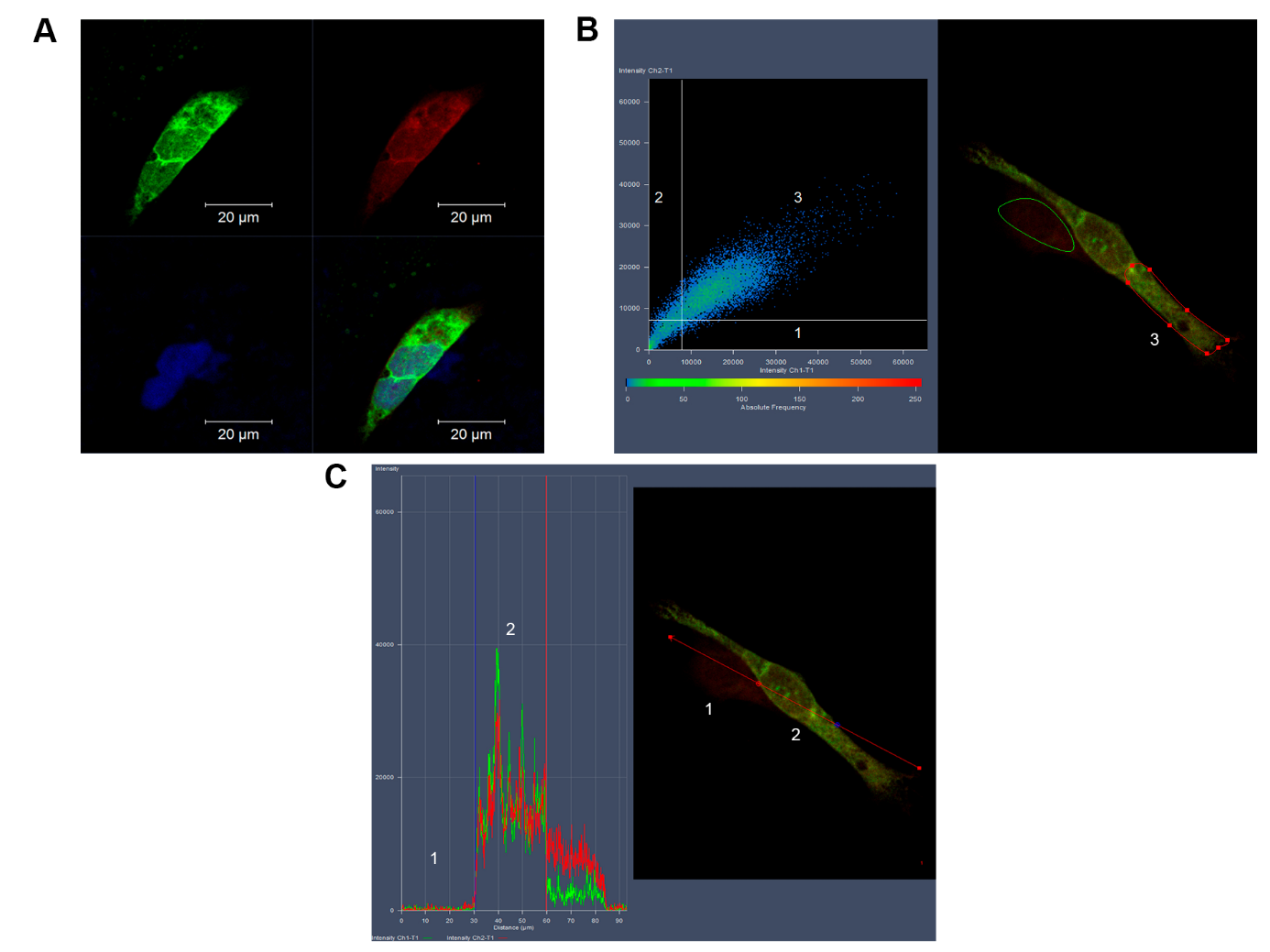


**Supplementary Figure 3.** (A) Paraformaldehyde fixed G01 cells, stained with anti-CD133/2-PE antibodies (Miltenyi Biotec, 130-113-748, Germany), and anti-GFP antibodies (Abcam Ab6662, UK). CD133 (red), GFP (green), DAPI (blue). (B) Colocalization of CD133CT and GFP in the fixed G01 cells. (C) Signal location along the axis (red line) through the areas of CD133CT localization (1, red) and CD133CT and GFP (2, red and green).

After transfection of cells with the CD133CT/pEGFP-c1 recombinant DNA, the cells were stained with anti-CD133/2-PE (phycoerythrin) antibodies (Miltenyi Biotec 130-113-748, Germany) and anti-GFP antibodies (Abcam Ab6662, UK), a membrane location of both proteins is seen (Supplementary Figure 3A). The diagram of intensity of signals from anti-CD133/2-PE antibodies and anti-GFP antibodies (Supplementary Figure 3B, left) shows that the cell is stained with both antibodies in the selected area 3. Supplementary Figure 3Cshows the same results: there is a united signal from both antibodies in the area 2 comparing to the area.


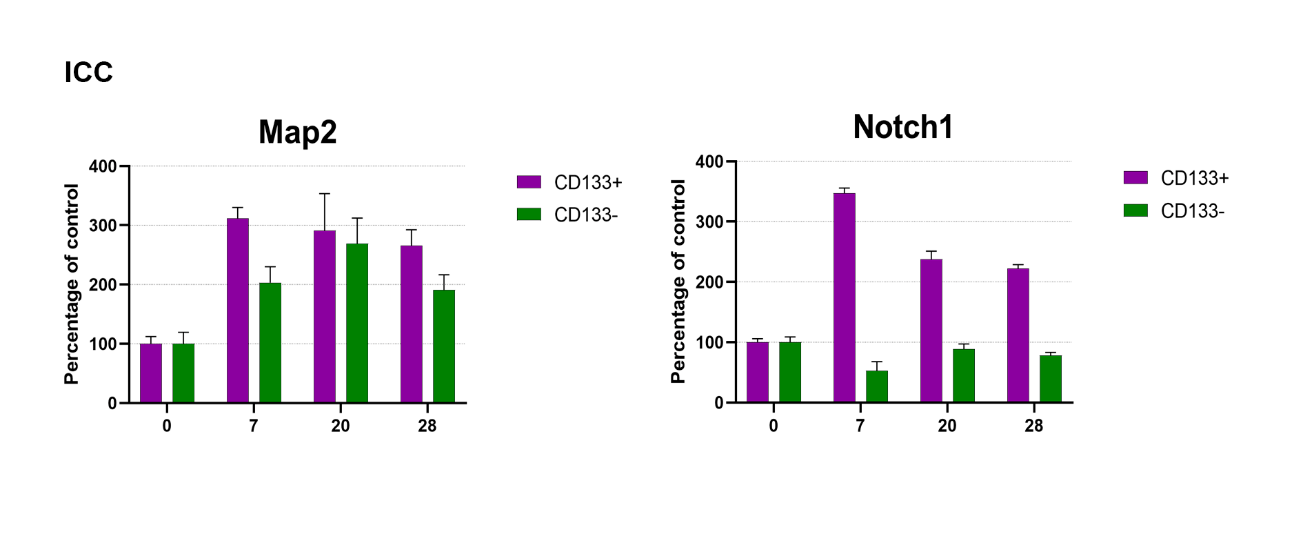


**Supplementary Figure 4.** Notch1+ cells in CD133+ and CD133– calculated from immunocytochemical staining with anti-Notch1 and anti-Map2 antibodies. Mean ± SD.


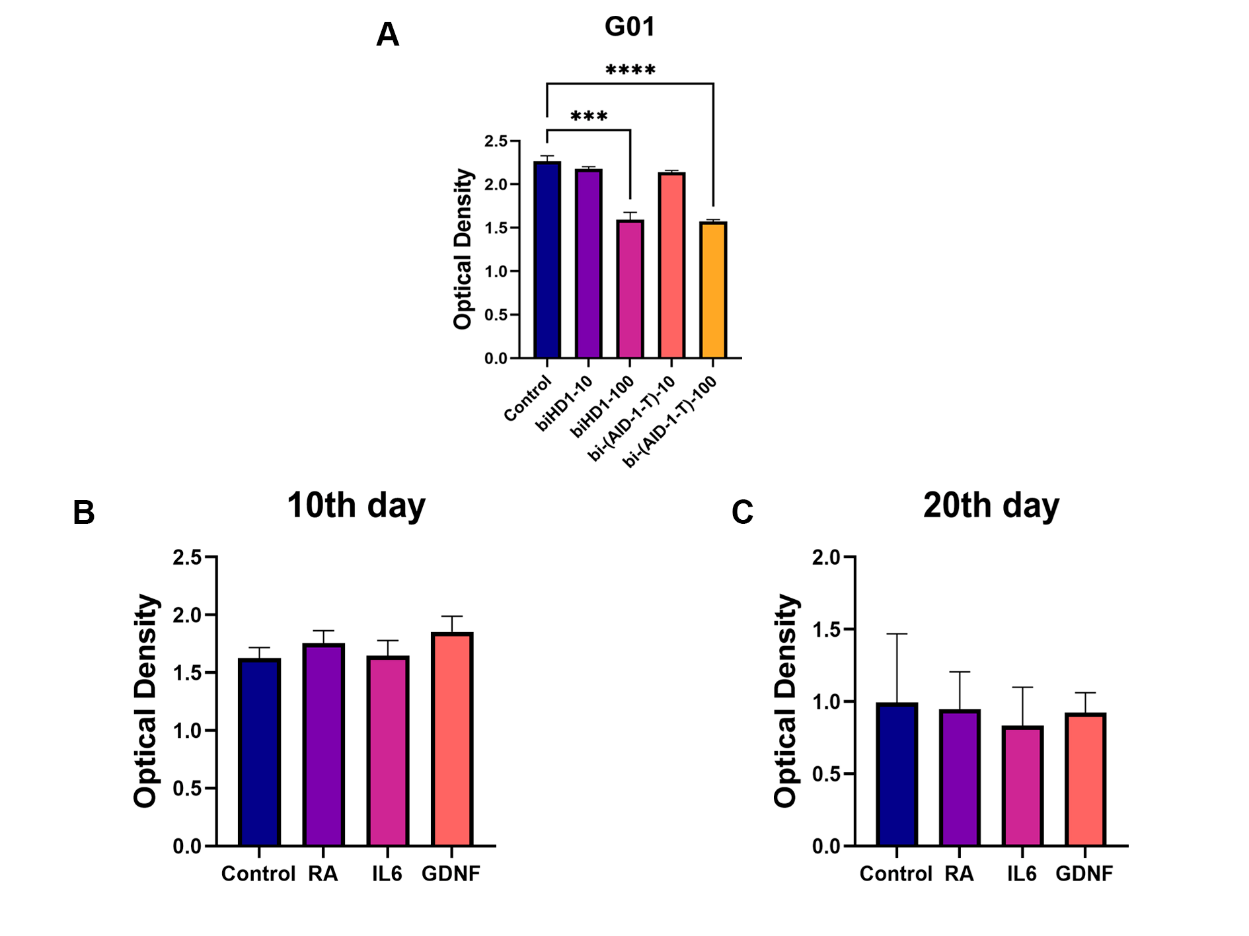


**Supplementary Figure 5.** MTT assay for GQ and factors. (A) 10 days after treatment with 10 µM and 100 µM biHD1 and bi-(AID-1-T); mean ± SD, n=5 for each group. MTT assay results after cells’ exposure to a mixture of aptamers (VEGF, as1411, r-21) and inducers of neural differentiation (retinoic acid, Il6 and GDNF in 10 days after exposure to differentiation inducers (B), by the day 20 days after exposure to differentiation inducers (C). Data are represented as mean ± SD. n=4 for each group. Statistically significant differences between the control and the treatment groups are indicated by asterisks (One-Way ANOVA, post-hoc Tukey HSD Test, *** = p<0.001, **** = p<0.0001). RA-retinoic acid.

**
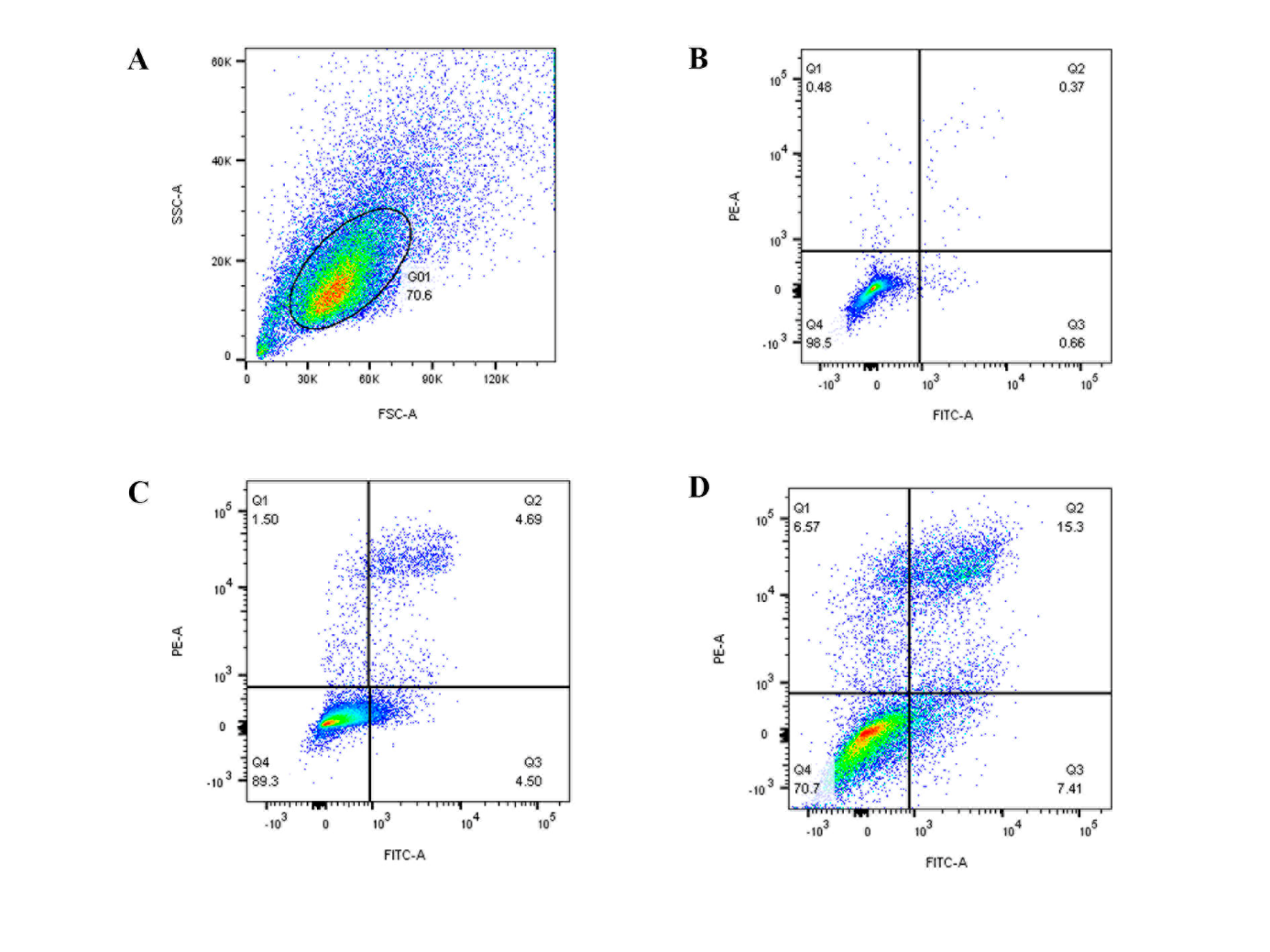
**

**Supplementary Figure 6.** Flow cytometry of G01 cells after exposure to GQIcombi. (A) Target population of untreated cells. (B) Apoptotic and necrotic cells in G01 control cells. Control G01 cells are localized in the Q4 region on a two-dimensional diagram of the dependence of the intensity of the fluorescent signals of fluorescein (apoptosis) and propidium-iodide (necrosis). (С) Apoptotic and necrotic cells in G01 cell culture incubated in the presence of biG3T. Cells in the Q3 region are associated with fluorescein-labeled annexin. (D) Apoptotic and necrotic cells in G01 cell culture incubated in the presence of biG3T and the cocktail of growth factors.

For untreated cells more than 70% of cells are included in a target population (Supplementary Figure 6A). When incubated in the presence of biG3T, the percentage of apoptotic cells is 4.50% of the total number of cells (Supplementary Figure 6C). When incubated in the presence of biG3T and a cocktail of growth factors the percentage of apoptotic cells is 7.14% of the total number of cells (Supplementary Figure 6D).

**
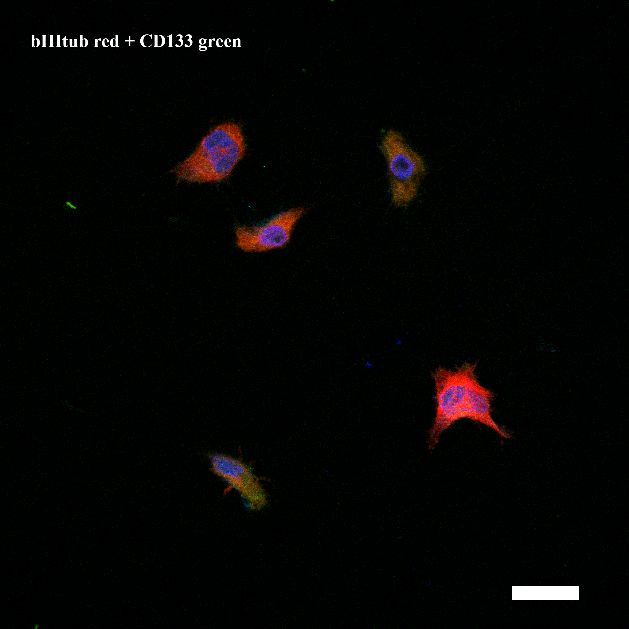
**

**Supplementary Figure 7.** Immunocytochemical staining with anti-bIII-tubulin antibodies (red) and anti-CD133 antibodies (green); scale bar is 20 μm.
